# Self-reported difficulty initiating sleep and early morning awakenings are associated with nocturnal diastolic non-dipping in older white Swedish men

**DOI:** 10.1038/s41598-020-70399-y

**Published:** 2020-08-07

**Authors:** Xiao Tan, Lars Lind, Martin Ingelsson, Johan Sundström, Jonathan Cedernaes, Christian Benedict

**Affiliations:** 1 Department of Neuroscience, Uppsala University, Box 593, 751 24 Uppsala, Sweden; 2 Department of Medical Sciences, Cardiovascular Epidemiology, Uppsala University, Uppsala, Sweden; 3 Department of Public Health and Caring Sciences, Uppsala University, Uppsala, Sweden; 4 Department of Medical Sciences, Clinical Epidemiology, Uppsala University, Uppsala, Sweden; 5 Department of Medical Sciences, Uppsala University, Uppsala, Sweden

**Keywords:** Biomarkers, Diagnostic markers, Cardiology

## Abstract

Chronically blunted nocturnal blood pressure (BP) dipping has been shown to increase the future risk of cardiovascular diseases. In the present cross-sectional study, we investigated whether self-reported insomnia symptoms were associated with an altered 24-h BP profile and blunted nocturnal BP dipping (night-to-day BP ratio > 0.90) in older men. For the analysis, we used 24-h ambulatory blood pressure data and reports of insomnia symptoms (difficulty initiating sleep, DIS; and early morning awakenings, EMA) from 995 Swedish men (mean age: 71 years). Compared to men without DIS, those reporting DIS (10% of the cohort) had a higher odds ratio of diastolic non-dipping (1.85 [1.15, 2.98], P = 0.011). Similarly, men who reported EMA (19% of the cohort) had a higher odds ratio of diastolic non-dipping than those without EMA (1.57 [1.09, 2.26], P = 0.015). Despite a slightly higher nocturnal diastolic BP among men with EMA vs. those without EMA (+ 1.4 mmHg, P = 0.042), no other statistically significant differences in BP and heart rate were found between men with and those without insomnia symptoms. Our findings suggest that older men reporting difficulty initiating sleep or early morning awakenings may have a higher risk of nocturnal diastolic non-dipping. Our findings must be replicated in larger cohorts that also include women.

## Introduction

Insomnia is a prevalent sleep problem in older population^1^. In addition to daytime impairment (e.g. sleepiness, being unfocused), at least one of the following symptoms characterizes insomnia: difficulty falling asleep, difficulty returning to sleep, and early morning awakenings^1^. Insomnia is associated with a multitude of health consequences, including hypertension^2^. For instance, a meta-analysis comprising 58,924 participants showed that chronic insomnia complaints were associated with an increased risk of hypertension, typically diagnosed by the auscultation method by using a mercury or automatic sphygmomanometer^3^. The association between insomnia symptoms and 24-h blood pressure (BP) profile has, however, been less studied. In a small study of middle-aged normotensive participants that measured blood BP over 24 h or longer, chronic insomnia was associated with both elevated nocturnal systolic BP and blunted nocturnal systolic BP dipping (typically defined as night-to-day BP ratio greater than 0.90^4^). Non-dippers have a higher risk of serious cardiac events than dippers, e.g. congestive heart failure^5,6^. Whether insomnia symptoms associate with an unhealthy 24-h blood pressure profile in older men is, however, not well researched. Aging increases the risk of insomnia^7^ and hypertension^8^.

Against this background, the present cross-sectional analysis involving 995 older men investigated whether reports of difficulty falling asleep and early morning awakening are associated with 24-h BP. Systolic and diastolic dipping may associate differently with cardiovascular outcomes. For instance, isolated diastolic but not isolated systolic non-dipping pattern has been related with arterial stiffness in young but not older adults^9^. Thus, we investigated associations of insomnia symptoms with both diastolic and systolic blood pressure 24-h profiles. Overall, we hypothesized that older men reporting difficulty initiating sleep and early morning awakenings have a higher relative risk of being a systolic and diastolic non-dipper than those without these insomnia symptoms.

## Methods

### Population and study design

The present cross-sectional study utilized variables from the age-70 investigation of the Uppsala Longitudinal Study of Adult Men (ULSAM; https://www.pubcare.uu.se/ulsam/). When started between September 1970 and September 1973, the main objective of ULSAM was to identify metabolic risk factors for cardiovascular disease in middle-aged men. A total of 2,322 white Swedish men living in Uppsala, Sweden (response rate: 82% of those invited) participated in the baseline investigation at age 50^10^. Although participants attended several consecutive follow-up investigations to measure a variety of behavioral and clinical parameters (e.g. lifestyle, clinical chemistry panels), assessments relevant for the present analysis only occurred at the follow-up investigation at age 70 (carried out between August 1991 and May 1995). Questionnaires utilized in the present study to assess, e.g., sleep had been filled out shortly before participants came to the test center to undergo physical investigations (e.g., blood pressure monitoring). From the 1,221 men who participated in the age 70-investigation, 18.5% were excluded because of missing data. Following these exclusions, 995 men (81.5%) were available for the final analysis. All participants gave written informed consent and the study was approved by the Regional Ethical Review Board in Uppsala (251/90 and 97/329). All research was performed in accordance with relevant guidelines/regulations.

### Insomnia symptoms

Participants answered the following questions: “Do you have difficulties falling asleep at night?”, (hereafter referred to as ‘difficulty initiating sleep’, DIS); and “Do you often wake up in early hours, unable to get back to sleep?”, (hereafter referred to as ‘early morning awakenings’, EMA). Participants could either answer ‘yes’, ‘no’, or “I don’t know” to these questions. The answer option “I don’t know” was treated as a missing variable. Note that no other questions related to sleep complaints, such as difficulty maintaining sleep and snoring were collected in ULSAM (see https://www.pubcare.uu.se/ulsam/).

### Prevalence of hypertension and ambulatory blood pressure monitoring (ABPM)

Hypertension was defined according to the 2018 guidelines of the European Society of Cardiology and the European Society of Hypertension as a 24-h average ambulatory systolic BP ≥ 130 mm Hg or a 24-h average ambulatory diastolic BP ≥ 80 mm Hg, and/or receiving antihypertensive drugs for treating hypertension^11^.

As described elsewhere^5^, the 24-h ambulatory BP and heart rate profiles were measured using Accutracker II (Suntech Medical Instruments, Raleigh, NC). The device was attached to participants’ non-dominant arm by an experienced healthcare technician. Systolic and diastolic BP, as well as heart rate, were measured every 20 or 30 min during daytime (06.00–23.00 h) and every 20 or 60 min during nighttime (23.00–06.00 h). Data were edited by omitting all readings presumed to be erroneous, including readings of 0, DBP readings of more than 170 mm Hg, SBP readings of more than 270 or less than 80 mm Hg, and all readings in which the difference between SBP and DBP was less than 10 mm Hg. The measurements were interpreted and the data edited by skilled laboratory technicians blinded to the study outcome. The Accutracker II device showed satisfactory accuracy and precision according to available documentation at the time of investigation in the early 1990s. As described elsewhere^12,13^, non-dipping was defined as nighttime:daytime BP ratio > 0.90. Reversed nocturnal BP dipping (nighttime:daytime BP ratio > 1.00) and extreme nocturnal BP dipping (nighttime: aytime BP ratio ≤ 0.80) patterns were regarded as non-dippers and dippers, respectively.

### Assessments of potential confounders

Body mass index (BMI) was defined as body weight (kg)/body height (m)^2^. Questionnaires were used to assess participants’ current smoking status (yes/no/not reported), level of leisure time physical activity (yes/no to the question “Do you do any active sport or heavy gardening for at least 3 h every week?”), and frequent use (> 3times/week) of hypnotics (yes/no to the question “Do you take sleeping pills more than 3 times a week?”). Note that we included the use of hypnotics as confounder in the analysis as it may mask reports of insomnia symptoms. Alcohol consumption (percentage of total daily calorie consumption) was assessed using the 7-day dietary history method. The presence of diabetes (treated as binary variable) was either confirmed by reviewing medical records, fasting plasma glucose ≥ 7.0 mmol/L, or when the 2 h oral glucose tolerance test (OGTT) blood glucose value and one or more of the 30–90 min OGTT plasma glucose values were ≥ 11.1 mmol/L^14^. Myocardial infarction (ICD 9 code 410 or ICD 10 code I21) and heart failure (ICD 9 code 428 or ICD 10 code I50) prior to the age 70 investigation were assessed from the hospital discharge register (available for all ULSAM participants). A detailed description of the methods that were used to assess covariates can be found at the ULSAM webpage: https://www.pubcare.uu.se/ulsam/.

### Statistical analysis

Analyses were performed with Stata 15.1 (StataCorp LLC, College Station, TX, USA). Normal distribution of continuous variables was confirmed by approximately symmetrical and bell-shaped histograms. Logarithmic transformation was performed to achieve normal distribution for nighttime systolic BP. One-way analyses of covariance (ANCOVA) were used to test possible differences in ambulatory BP and heart rate variables between men with and without insomnia symptoms. Logistic regression was performed to examine the association between insomnia symptoms, non-dipping, and hypertension. In the basic model, we adjusted for participants’ age. The advanced model further included BMI, hypertension status, hypertension treatment, diabetes status, smoking status, alcohol consumption, leisure time physical activity, history of myocardial infarction or heart failure, and reports of frequent use of hypnotics. Note that our assumptions regarding dependencies between predictor, outcome, and confounder variables were based on data from the published literature^15–18^. Overall, a two-sided P value of less than 0.05 was regarded as statistically significant.

## Results

### Cohort characteristics

Descriptive data of the cohort are summarized in Table 1. Difficulty initiating sleep (DIS) was reported by 10% of the men. Early morning awakenings (EMA) were reported by 19% of the men. Sixty-nine percent of the study population (n = 686) had hypertension, and about 30% of the participants were classified as non-dippers (i.e., nocturnal fall in BP was < 10%). Both systolic and diastolic non-dipping were associated with increased odds of hypertension (OR, 95% CI systolic non-dippers vs. systolic dippers, 1.64 [1.21, 2.21], P = 0.001; diastolic non-dippers vs. diastolic dippers, 1.50 [1.10, 2.04], P = 0.011; each adjusted for age).

**Table 1:** Characteristics of the study population (995 older white Swedish men), divided by insomnia complaints.

Parameter	Total cohort	DIS		EMA	
	N = 995	Yes (N = 103)	No (N = 892)	Yes (N = 190)	No (N = 805)
Age (years)	71.0 ± 0.6	71.0 ± 0.6	71.0 ± 0.6	71.0 ± 0.6	71.0 ± 0.6
BMI (kg/m^2^)	26.2 ± 3.4	26.0 ± 4.0	26.3 ± 3.3	26.8 ± 3.8	26.1 ± 3.3
**Mean ambulatory systolic BP (mmHg)**					
24-h	135 ± 16	134 ± 14	135 ± 16	136 ± 15	135 ± 16
0,600–2,300 h	139 ± 16	137 ± 14	139 ± 16	139 ± 15	139 ± 16
2,300–0,600 h	121 ± 19	121 ± 17	121 ± 19	123 ± 17	120 ± 19
**Mean ambulatory diastolic BP (mmHg)**					
24-h	76 ± 8	76 ± 8	76 ± 8	77 ± 8	76 ± 8
0,600–2,300 h	79 ± 8	78 ± 8	79 ± 8	79 ± 8	79 ± 8
2,300–0,600 h	68 ± 9	69 ± 9	67 ± 9	69 ± 9	67 ± 9
**Mean ambulatory heart rate (bpm)**					
24-h	69 ± 10	70 ± 9	69 ± 10	69 ± 10	69 ± 10
0,600–2,300 h	71 ± 11	72 ± 10	71 ± 11	71 ± 10	71 ± 11
2,300–0,600 h	61 ± 9	62 ± 8	61 ± 10	61 ± 9	61 ± 10
**Non-dipping, N (%)**					
Systolic	323 (32)	41 (40)	282 (32)	69 (36)	254 (32)
Diastolic	283 (28)	43 (42)	240 (27)	69 (36)	214 (27)
**Reverse dipping, N (%)**					
Systolic	56 (6)	9 (9)	47 (5)	14 (7)	42 (5)
Diastolic	45 (5)	6 (6)	39 (4)	8 (4)	37 (5)
**Extreme dipping, N (%)**					
Systolic	196 (20)	14 (14)	182 (20)	30 (16)	166 (21)
Diastolic	236 (24)	27 (26)	209 (23)	40 (21)	196 (24)
**Hypertension status, N (%)**					
Yes	686 (69)	70 (68)	616 (69)	135 (71)	551 (68)
No	309 (31)	33 (32)	276 (31)	55 (29)	254 (32)
**Hypertension treatment, N (%)**					
Yes	353 (35)	39 (38)	314 (35)	64 (34)	289 (36)
No	642 (65)	64 (62)	578 (65)	126 (66)	516 (64)
**Diabetes status, N (%)**					
Yes	145 (15)	19 (18)	126 (14)	34 (18)	111 (14)
No	850 (85)	84 (82)	766 (86)	156 (82)	694 (86)
**Smoking status, N (%)**					
Yes	194 (19)	33 (32)	161 (18)	26 (14)	168 (21)
No	784 (79)	68 (66)	716 (80)	158 (83)	626 (78)
Not reported	17 (2)	2 (2)	15 (2)	6 (3)	11 (1)
**Alcohol consumption (E%)**	2.7 ± 3.1	2.5 ± 3.0	2.7 ± 3.1	2.5 ± 3.2	2.8 ± 3.1
**Leisure time physical activity ≥ 3 h/week**					
Yes	613 (62)	55 (53)	558 (63)	107 (56)	506 (63)
No	382 (38)	48 (47)	334 (37)	83 (44)	299 (37)
**History of MI/HF, N (%)**					
Yes	63 (6)	9 (9)	54 (6)	13 (7)	50 (6)
No	932 (94)	94 (91)	838 (94)	177 (93)	755 (94)
**Use of hypnotics > 3 times/week, N (%)**					
Yes	40 (4)	24 (23)	16 (2)	26 (14)	14 (2)
No	949 (95)	77 (75)	872 (98)	159 (84)	790 (98)
Not reported	6 (1)	2 (2)	4 (0)	5 (3)	1 (0)

Data are presented as mean ± SD, unless otherwise specified. Non-dipping: night-to-day BP ratio > 0.90; reversed dipping: night-to-day BP ratio > 1.00; extreme dipping: night-to-day BP ratio ≤ 0.80.

*BMI* body mass index, *BP* blood pressure, *DIS* difficulty initiating sleep, *EMA* early morning awakening, *E%* percentage of total daily calorie consumption, *HF* heart failure, *MI* myocardial infarction.

### Association of insomnia symptoms with blood pressure and heart rate

Utilizing ANCOVA, a significantly higher nighttime diastolic BP was found for men with EMA, compared with those without reports of EMA (+ 1.4 mmHg, P = 0.042; Table 2). No other significant group differences in BP and heart rate were found (P > 0.09 for all ANCOVA comparisons, Table 2).

**Table 2:** Main associations between insomnia symptoms and blood pressure or heart rate.

Analysis	Dependent variable	Difficulty initiating sleep (DIS) vs. no DIS				Early morning awakenings (EMA) vs. no EMA			
		Basic model		Advanced model		Basic model		Advanced model	
		Adjusted mean difference/OR (95% CI)	P	Adjusted mean difference/OR (95% CI)	P	Adjusted mean difference/OR (95% CI)	P	Adjusted mean difference/OR (95% CI)	P
	**Systolic BP (mmHg)**								
ANCOVA	24-h	− 1.30 (− 4.57, 1.97)	0.44	− 0.98 (− 3.71, 1.76)	0.48	0.83 (− 1.71, 3.37)	0.52	0.46 (− 1.59, 2.52)	0.66
	06.00–23.00 h	− 1.77 (− 5.06, 1.53)	0.29	− 1.39 (− 4.19, 1.40)	0.33	0.63 (− 1.93, 3.18)	0.63	0.20 (− 1.90, 2.30)	0.85
	23.00–06.00 h^a^	0.67 (− 3.15, 4.49)	0.81	0.80 (− 2.79. 4.39)	0.53	2.24 (− 0.72, 5.19)	0.13	1.89 (− 0.80, 4.58)	0.10
	**Diastolic BP (mmHg)**								
ANCOVA	24-h	0.00 (− 1.61, 1.61)	0.99	0.25 (− 1.27, 1.77)	0.75	0.55 (− 0.70, 1.80)	0.39	0.72 (− 0.42, 1.85)	0.22
	06.00–23.00 h	− 0.35 (− 2.02, 1.32)	0.68	− 0.10 (− 1.70, 1.51)	0.91	0.41 (− 0.89, 1.70)	0.54	0.58 (− 0.62, 1.79)	0.34
	23.00–06.00 h	1.47 (− 0.32, 3.25)	0.11	1.38 (− 0.41, 3.17)	0.13	**1.41 (0.03, 2.79)**	**0.045**	**1.39 (0.05, 2.74)**	**0.042**
	**Heart rate (bpm)**								
ANCOVA	24-h	0.35 (− 1.70, 2.41)	0.74	− 0.43 (− 2.65, 1.79)	0.70	− 0.10 (− 1.69, 1.50)	0.91	0.32 (− 1.35, 1.98)	0.71
	06.00–23.00 h	0.15 (− 2.02, 2.33)	0.89	− 0.51 (− 2.87, 1.85)	0.67	− 0.16 (− 1.85, 1.52)	0.85	0.33 (− 1.44, 2.10)	0.71
	23.00–06.00 h	0.69 (− 1.22, 2.61)	0.48	− 0.46 (− 2.52, 1.59)	0.66	0.31 (− 1.17, 1.80)	0.68	0.59 (− 0.95, 2.13)	0.45
	**Non-dipping**								
LoR	Systolic	1.43 (0.94, 2.17)	0.10	1.46 (0.91, 2.35)	0.12	1.24 (0.89, 1.72)	0.21	1.36 (0.95, 1.95)	0.09
	Diastolic	**1.95 (1.28, 2.96)**	**0.002**	**1.85 (1.15, 2.98)**	**0.011**	**1.57 (1.13, 2.20)**	**0.008**	**1.57 (1.09, 2.26)**	**0.015**

Non-dipping was defined as night-to-day BP ratio > 0.90. *Basic model*: adjusted for age. *Advanced model*: adjusted for age, BMI, hypertension status, hypertension treatment, diabetes status, smoking status, alcohol consumption, leisure-time physical activity, history of myocardial infarction or heart failure, and frequent use of hypnotics. A P-value (P) smaller than 0.05 was considered significant (shown in bold).

*BMI* body mass index, *BP* blood pressure, *bpm* beta per minute, *DIS* difficulty initiating sleep, *EMA* early morning awakening, *LoR* logistic regression.

^a^Comparisons under log-transformed values.

### Association between insomnia symptoms and non-dipping

Logistic regression analyses demonstrated that men with DIS had an 85% (95% CI 15%, 298%) higher odds ratio of being a diastolic non-dipper than those without reports of DIS (Table 2). We also found that men with EMA had a 57% (95% CI 9–226%) higher odds ratio of being a diastolic non-dipper, compared to men without EMA. Both DIS and EMA were also associated with a higher odds ratio for systolic non-dipping; however, these associations did not reach significance (Table 2).

### Sensitivity analyses

Anti-hypertensive medications, and diuretics in particular, may contribute to nocturia, which in turn would precipitate symptoms of insomnia. Thus, we ran a sensitivity analysis excluding participants with antihypertensive treatment. This sub-group analysis produced similar results [night-time diastolic BP, EMA vs. no EMA: P = 0.10; OR for diastolic non-dipping (95% CI), DIS vs. no DIS: 1.92 (1.07, 3.45), P = 0.029; EMA vs. no EMA: 1.69 (1.07, 2.65), P = 0.023, all in fully adjusted model].

## Discussion

The present study involved 995 men aged 71 years in whom we examined whether reports of difficulty initiating sleep (DIS) and early morning awakenings (EMA) were associated with an altered 24-h blood pressure (BP) profile. Our main finding was that men reporting either EMA or DIS had a higher relative risk to suffer from nocturnal diastolic non-dipping (i.e., night-day BP ratio greater than 0.90) than diastolic dippers. Essentially the same results were obtained when excluding those with antihypertensive treatment.

As of yet, the association between insomnia symptoms and 24-h ambulatory blood pressure patterns has not been well researched, especially in older populations. One small study showed that middle-aged patients with chronic primary insomnia (n = 13, mean age: 42 ± 7 years) had higher nighttime systolic BP and blunted nocturnal systolic BP dipping, compared with controls (n = 13)^4^. By utilizing 24-ABPM measurements and objective and subjective markers of insomnia, a separate study involving 502 participants aged 30–60 years from the Wisconsin sleep cohort documented significant cross-sectional associations of subjective and objective sleep quality markers with BP non-dipping (including both systolic and diastolic non-dipping). Additionally, the same study showed in longitudinal analyses that longer wake after sleep onset, longer total sleep time and lower sleep efficiency were associated with higher risk of systolic non-dipping^19^. In contrast, our study did not find differences in systolic dipping between older men with and without insomnia symptoms. These disparaging findings between the Wisconsin sleep cohort and the ULSAM cohort may be due to the differences in age, sex (the Wisconsin study included both men and women whereas only men were included in the present study), selection of confounders, and the method to determine insomnia symptoms. Finally, we cannot rule out the possibility that our cohort was too small to detect a significant association between self-reported insomnia symptoms and 24-h systolic BP patterns.

Our observational study is limited as we cannot establish causality. There are, however, several potential mechanisms that may explain the association between insomnia symptoms and diastolic BP non-dipping, such as increased nocturnal sympathetic nervous system activity^20^ and repeated change in body posture during the night (e.g. repeated transition from supine to upright body posture because of an inability to fall asleep).

Whatever the precise mechanism underlying the association between insomnia symptoms and nocturnal diastolic non-dipping, there are several important considerations that should be taken into account when interpreting the results of our cross-sectional study. First, we did not investigate whether other potential exposure pathways related to BP and hypertension, such as socioeconomic status^21^, may explain the observed difference in diastolic non-dipping between older men with and without insomnia symptoms. Another limitation of our study is the lack of information on the prevalence of obstructive sleep apnea (OSA) or short sleep duration. OSA and short sleep duration have been linked to both cardiovascular problems^22,23^ and non-dipping^24,25^. Those experiencing OSA or short sleep duration also often report symptoms of insomnia^26,27^. Thus, OSA or habitual short sleep duration may partially explain the association between insomnia symptoms and diastolic non-dipping in the present study. Another limitation is that no data on individuals’ habitual sleep onset and offset were available. Thus, we could not match BP data with individual sleep (rest) and wake (activity) cycles. Another limitation of our study is that the number of blood pressure cuff inflations varied between once or three times per hour during the night. Thus, it is possible that a resulting difference in sleep quality may have influenced the results.

In our study, we investigated the association of two major insomnia symptoms with the 24-h blood pressure profile in older men. We did not have information about the frequency and duration of the assessed insomnia symptoms, nor did the participants provide reports about difficulty maintaining sleep or daytime symptoms (e.g. difficulty paying attention, focusing on tasks or remembering). Thus, our results do not allow any conclusion on how the sleep disorder insomnia would associate with the 24-h blood pressure profile in older men.

Epidemiological studies have shown that impaired breathing patterns during sleep are associated with a significantly increased odds ratio for hypertension in men but not women^28^. In a separate study, habitual short duration of sleep (defined as ≤ 5 h per night) was associated with higher risk of hypertension compared with the group sleeping 7 h, among women but not men^29^. Finally, a study involving more than 700,000 adults found for both men and women, the relative risk of having hypertension associated with short sleep decreased with increasing age, but there was a higher association between short sleep and hypertension for women, throughout the adult lifespan^30^. With all this in mind, our findings should not be generalized to women and other ages.
